# Differential responses of *Trans*-Resveratrol on proliferation of neural progenitor cells and aged rat hippocampal neurogenesis

**DOI:** 10.1038/srep28142

**Published:** 2016-06-23

**Authors:** Vivek Kumar, Ankita Pandey, Sadaf Jahan, Rajendra Kumar Shukla, Dipak Kumar, Akriti Srivastava, Shripriya Singh, Chetan Singh Rajpurohit, Sanjay Yadav, Vinay Kumar Khanna, Aditya Bhushan Pant

**Affiliations:** 1 System Toxicology and Health Risk Assessment Group, CSIR-Indian Institute of Toxicology Research (CSIR-IITR), MG Marg, Lucknow, Uttar Pradesh-226001, India; 2 Academy of Scientific & Innovative Research, CSIR-IITR Campus, Lucknow, India

## Abstract

The plethora of literature has supported the potential benefits of Resveratrol (RV) as a life-extending as well as an anticancer compound. However, these two functional discrepancies resulted at different concentration ranges. Likewise, the role of Resveratrol on adult neurogenesis still remains controversial and less understood despite its well documented health benefits. To gather insight into the biological effects of RV on neurogenesis, we evaluated the possible effects of the compound on the proliferation and survival of neural progenitor cells (NPCs) in culture, and in the hippocampus of aged rats. Resveratrol exerted biphasic effects on NPCs; low concentrations (10 μM) stimulated cell proliferation mediated by increased phosphorylation of extracellular signal-regulated kinases (ERKs) and p38 kinases, whereas high concentrations (>20 μM) exhibited inhibitory effects. Administration of Resveratrol (20 mg/kg body weight) to adult rats significantly increased the number of newly generated cells in the hippocampus, with upregulation of p-CREB and SIRT1 proteins implicated in neuronal survival and lifespan extension respectively. We have successfully demonstrated that Resveratrol exhibits dose dependent discrepancies and at a lower concentration can have a positive impact on the proliferation, survival of NPCs and aged rat hippocampal neurogenesis implicating its potential as a candidate for restorative therapies against age related disorders.

Neural progenitor cells (NPCs) are defined as self-renewing, multipotent cells that generate neurons, astrocytes, and oligodendrocytes in the nervous system^1^. NPCs are known to be responsive to several types of environmental stimuli including dietary restriction^2^,^3^, physical exercise^4^,^5^ and injury^6^,^7^ by increasing their proliferation and/or the survival of newly generated neurons. However with age neurons show a decreased capacity to compensate for any imbalance or injury. Moreover there is a close relationship between the onset of ageing and the appearance of neurodegenerative disorders that can lead to irreversible injuries to the brain. So, a better understanding of the factors that influence adult neurogenesis, and the underlying cellular and molecular mechanisms may lead to the development of restorative therapies for CNS injury and neurodegenerative disorders^8^.

Evidence from epidemiological and experimental studies that natural edible products can protect against diseases is currently best exemplified by Resveratrol (RV) (3,4′,5trihydroxystilbene), a polyphenolic phytoalexin that is abundant in several plants, and is found in grapes, peanuts, mulberries, pines and red wine^9^. The initial impetus for research on RV came from the paradoxical observation of low incidence of cardio-vascular diseases that coexist with intake of a high-fat diet and moderate consumption of red-wine in certain populations, a phenomenon known as French paradox^10^,^11^. Several studies within the last few years have exhibited pleiotropic health benefits of RV that it may prevent or slow the progression of a variety of human diseases, including cardio-vascular diseases^12^, cancer^13^ and ischemic injuries^14^ as well as enhance stress resistance^15^. It is also known to mitigate the metabolic dysfunction of mice fed with high-fat diets^16^.

More recent results provide interesting insights into the effect of RV on the life span of yeasts and flies, suggesting it’s potential as an anti-ageing agent in treating age-related human diseases^17^,^18^. In addition, it has been reported that RV can protect neurons against degeneration and dysfunction in experimental models of ischemic stroke, Alzheimer disease (AD), and Parkinson disease (PD)^19^. This increasing amount of convincing data have triggered an impressive plethora of studies investigating the potential of RV as a therapeutic strategy for treating age related disorders and its involvement in ageing.

Despite of well-documented health benefits, mechanism of action of RV remains largely controversial. The objective here is to clarify the intracellular mechanisms involved in mediating RV’s beneficial effects on the proliferation of embryonic rNPCs (rat neural progenitor cells) at different concentrations and also investigate hippocampal neurogenesis in aged rats. Emerging evidence suggests that some phytochemicals exert their beneficial effects via adapting a cellular stress response pathway^20^. To obtain insights into the molecular mechanism of RV’s mitogenic effect, we probed the MAPK (Mitogen Activated Protein Kinase) signalling pathway for their potential involvement. These signalling pathways were selected for our investigations because they are among the best known players involved in the regulation of cell growth and survival^21^,^22^. We therefore undertook this study to determine if and how RV might influence proliferation of embryonic NPCs and affect adult neurogenesis.

## Results

### Characterization and proliferation of rNPCs

**Figure 1:**
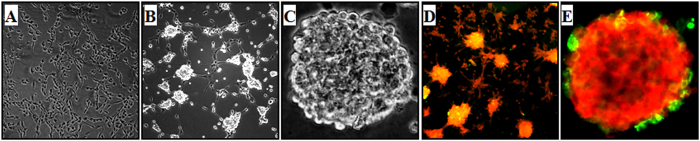
Characterization and proliferation of rNPCs. (**A**) Rat brain NPCs isolated from embryonic day-12 (ED-12) rat foetuses showing over 95% viability. (**B**) Proliferative cells seen as small neurospheres by day 7 in serum free neurobasal medium supplemented with growth factors. (**C**) The neurospheres attained maturity by day 20. (**D**,**E)** Representative micrographs showing expression of both progenitor cell marker-nestin (green) and proliferating cell marker-BrdU (red).

Rat brain NPCs isolated from embryonic day-12 (ED-12) rat foetuses showed over 95% viability (Fig. 1A). The proliferative cells were able to develop the small neurospheres by day 7 in serum free neurobasal medium supplemented with growth factors (Fig. 1B). The neurospheres attained maturity by day 20 (Fig. 1C). Cells in neurospheres showed expression of both progenitor cell marker-nestin (green) and proliferating cell marker-BrdU (red) (Fig. 1D,E).

### Lower concentration of RV stimulates proliferation of NPCs

**Figure 2:**
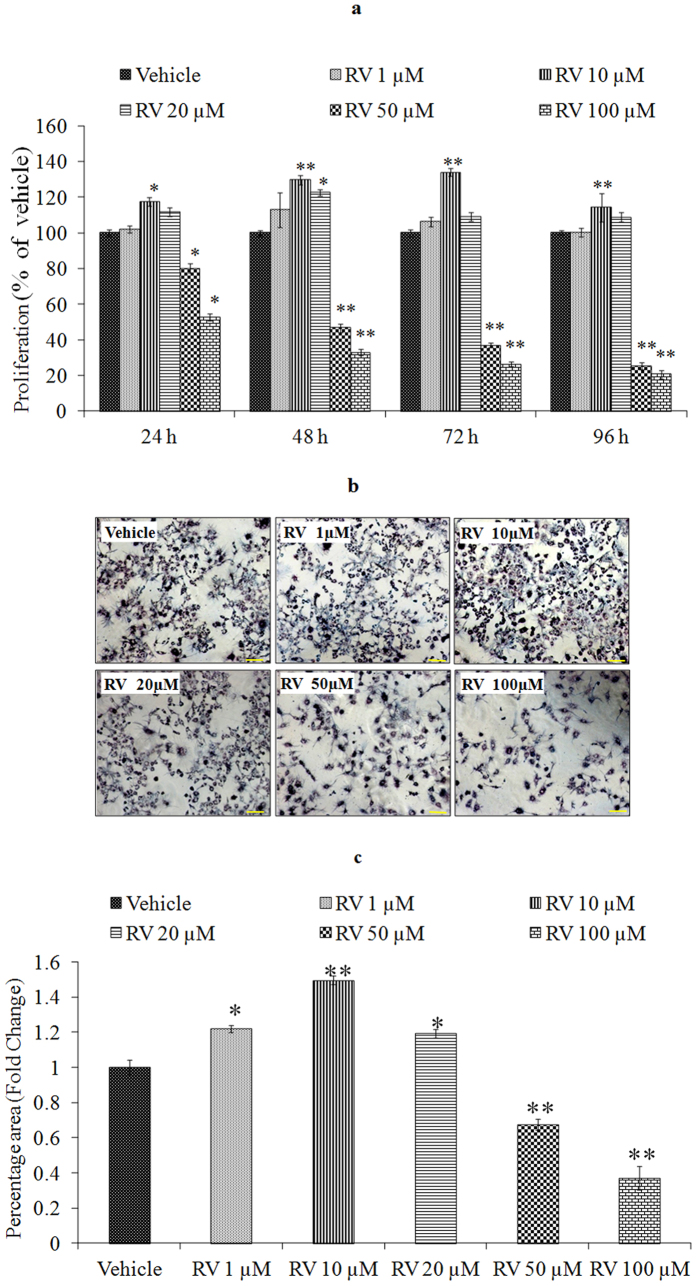
Lower concentrations of Resveratrol stimulates the proliferation of Neural Progenitor cells. (**a**) Cell viability assay of NPCs at 24–96 h following the exposure of different concentrations of Resveratrol by MTT assay. (**b**) Cell viability assay of NPCs by formazon crystal formation at 24 h following the exposure of different concentrations of Resveratrol by MTT assay. Images of formazon crystal were taken after exposure to MTT solution for 4 h. (**c**) Image quantification was done using ImageJ image analysis software and expressed in fold change. *p < 0.05; **p < 0.01; ***p < 0.001.

**Figure 3:**
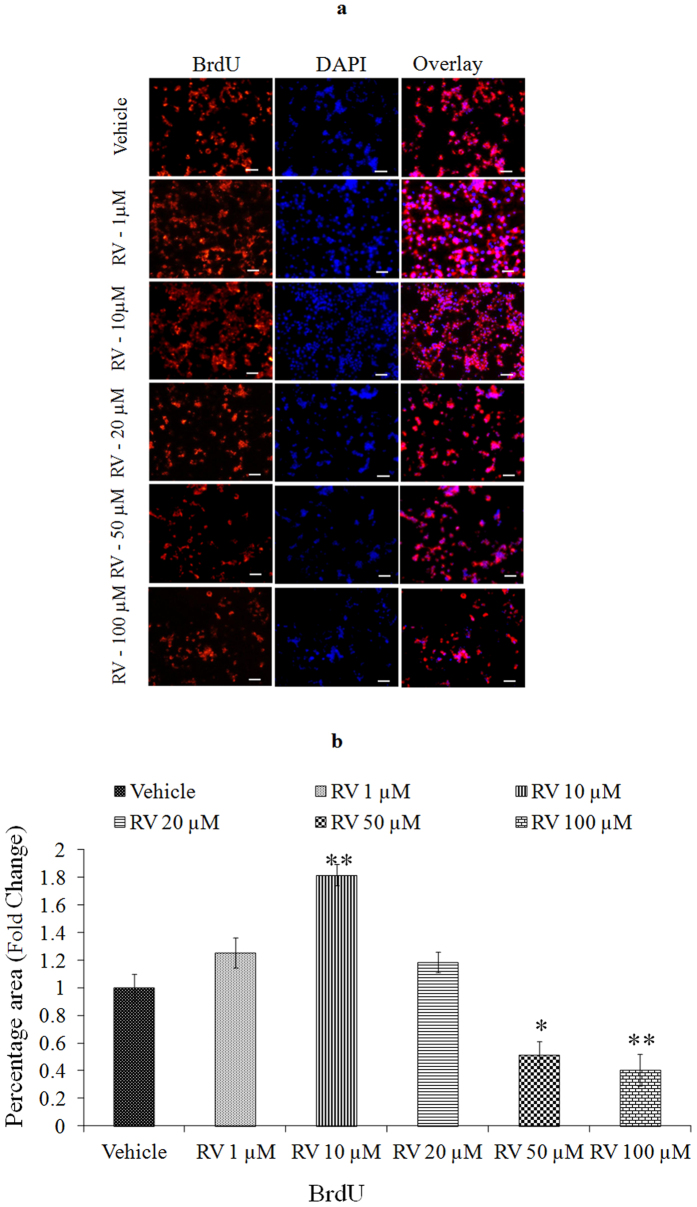
Stimulatory effects of lower concentrations of Resveratrol as confirmed by BrdU labelling. (**a**) BrdU immunoreactivity (red) in NPCs treated with 20 μM BrdU for 2 h and then exposed to Resveratrol for 24 h. Nuclei were counter stained with DAPI (blue). (**b**) Image quantification was done using ImageJ image analysis software and expressed in fold change. *p < 0.05; **p < 0.01; ***p < 0.001.

**Figure 4:**
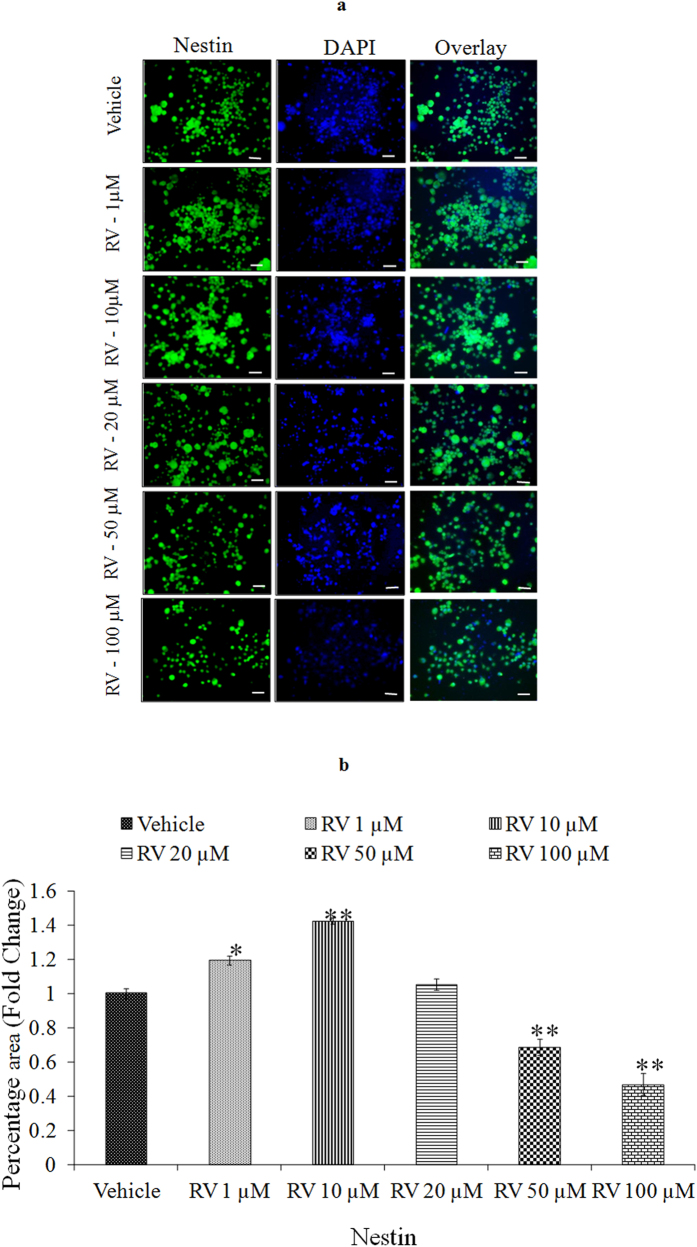
Stimulatory effects of lower concentrations of Resveratrol as confirmed by immunocytochemistry. (**a**) Representative microphotographs showing Immunocytochemistry localization of neural progenitor cell marker viz. Nestin (green) in NPCs following the exposure of Resveratrol for 24 h. (**b**) Image quantification was done using ImageJ image analysis software and expressed in fold change. *p < 0.05; **p < 0.01; ***p < 0.001.

**Figure 5:**
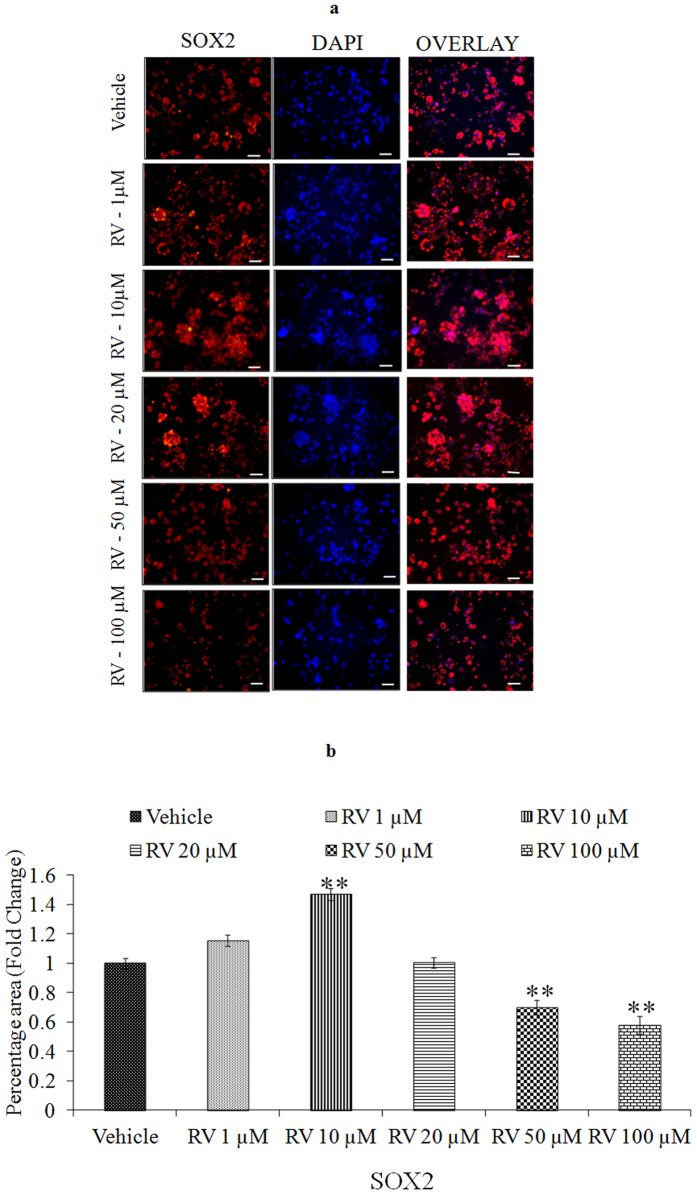
Stimulatory effects of lower concentrations of Resveratrol as confirmed by immunocytochemistry. (**a**) Representative microphotographs showing Immunocytochemistry localization of neural progenitor cell marker viz. SOX2 (red) in NPCs following the exposure of Resveratrol for 24 h. (**b**) Image quantification was done using ImageJ image analysis software and expressed in fold change. *p < 0.05; **p < 0.01; ***p < 0.001.

**Figure 6:**
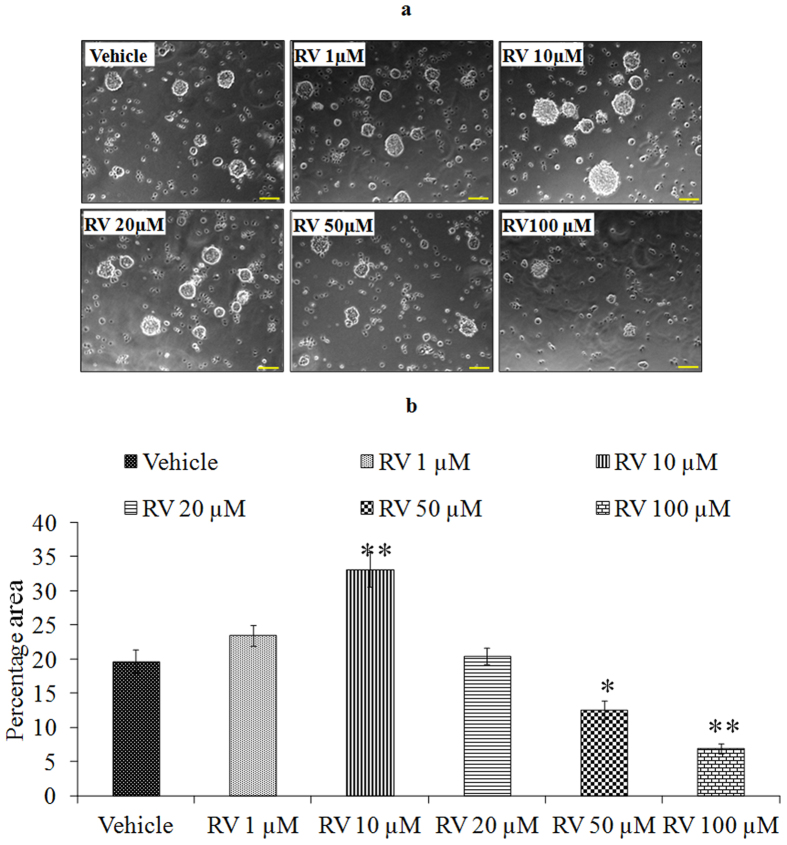
Lower concentrations of Resveratrol exposure resulted in increase in the number and size of neurospheres. (**a**) Embryonic neurospheres were grown in vehicle and different concentration of Resveratrol containing medium for 96 h. Expansion of neurospheres was observed in response to 10 μM of Resveratrol. (**b**) Image quantification was done using ImageJ image analysis software and expressed in fold change. *p < 0.05; **p < 0.01; ***p < 0.001.

**Figure 7:**
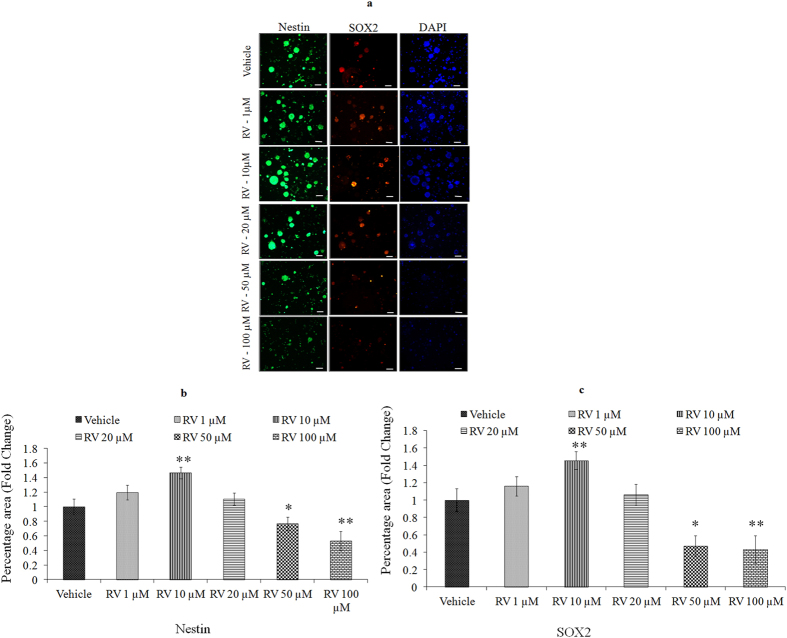
Lower concentrations of Resveratrol increase the expression of proliferation markers. (**a**) Represen-tative microphotographs showing Immunocytochemical localization of neural progenitor cell marker viz. Nestin (green) and SOX2 (red) in neurospheres following the exposure of Resveratrol for 96 h. The images were snapped by Nikon DS-Ri1 (12.7megapixel) camera using upright phase contrast florescence microscope (Nikon 80i, Japan) at ×10 × 10 magnification. Data represents mean ± SE of three independent experiments. *p < 0.05; **p < 0.01; ***p < 0.001 (Vehicle vs experimental group). Image quantification (**b)** Nestin, (**c**) SOX2 was done using ImageJ image analysis software and expressed in fold change.

We first performed a concentration and time-response experiment to determine whether RV modifies the proliferation of NPCs. The proliferative effects of low concentrations of RV, evaluated during a 4-day exposure period, revealed that the proliferation-promoting action of RV was progressively increased during the 4-day time period (Fig. 2a). After 24 h of exposure, lower doses (1, 10, 20 μM) increased NPC proliferation, whereas higher doses (50, 100 μM) decreased the proliferation in NPCs (Fig. 2b,c). Among the concentrations tested, 10 μM was the most effective in stimulating NPCs proliferation. To confirm the proliferative effect of RV, markers of proliferation such as BrdU labeling, Nestin and SOX2 were studied in NPCs using immuno-cytochemistry. Quantification of immune stained cells with BrdU (Fig. 3a,b), Nestin (Fig. 4a,b) and SOX2 (Fig. 5a,b) showed a significant increase in NPC proliferation in cultures exposed to 10 μM of RV for 24 h. We also checked the proliferative effect of RV in primary embryonic neurospheres. Neurospheres were exposed to different concentrations of RV (1–100 μM) in neurobasal medium for 96 h, analysis of neurospheres was done by Image J software. These results indicate that lower concentrations of RV (1, 10, 20 μM) exposure resulted in an increase in the number and size of neurospheres. In the range of lower concentrations tested, 10 μM was the most effective in stimulating neurospheres proliferation (Fig. 6a,b). However, higher concentration of RV (50–100 μM) significantly inhibited the number and size of neurospheres. In addition, we also carried out immunostaining studies to investigate the proliferation expression markers such as Nestin and SOX2 in neurospheres following the exposure to RV for 96 h. Quantification of immunostained neurospheres with Nestin and SOX2 showed a significant increase in the expression of these markers in cultures exposed to RV (10 μM) (Fig. 7a–c). These findings indicate that lower concentrations of RV induce proliferation, while higher doses of RV are cytotoxic.

### Lower concentrations of RV induces MAPK signalling molecules activation in NPCs

**Figure 8:**
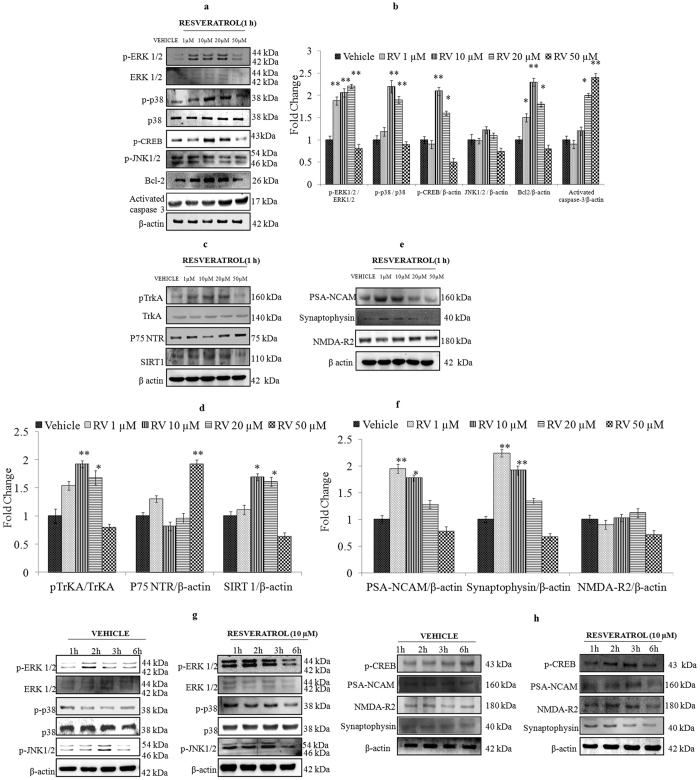
Low concentrations of Resveratrol activates ERK1/2 and p38 signalling molecules in NPCs. (**a**) Activation of the ERK1/2, p38 and JNK1/2, p-CREB, Bcl-2 and activated caspase-3 on exposure to different concentration of Resveratrol for 1 h. (**b**) Quantification was done in Gel Documentation System (Alpha Innotech, USA) with the help of AlphaEaseTM FC Stand-Alone V.4.0 software and expressed in fold change. (**c**) Activation of pTrKA, p75NTR, SIRT 1 on exposure to different concentrations of Resveratrol for 1 h. (**d**) Quantification was done in Gel Documentation System (Alpha Innotech, USA) with the help of AlphaEaseTM FC Stand-Alone V.4.0 software and expressed in fold change. (**e**) Activation of downstream genes of p-CREB viz. PSA-NCAM, Synaptophysin and NMDA R2 on exposure of Resveratrol for 1 h. (**f**) Quantification was done in Gel Documentation System (Alpha Innotech, USA) with the help of AlphaEaseTM FC Stand-Alone V.4.0 software and expressed in fold change. (**g**) Activation of the ERK1/2, p38 and JNK1/2 signalling molecules phosphorylation on exposure of 10 μM of Resveratrol for different time periods (1–6 h). (**h**) Activation of p-CREB, PSA-NCAM, Synaptophysin and NMDA R2 proteins on exposure of 10 μM Resveratrol for different time periods (1-6 h)β-actin was used as an internal control to normalize the data. *p < 0.05; **p < 0.01; ***p < 0.001.

MAPK pathways regulate cellular processes such as proliferation, survival/apoptosis, differentiation, development, adherence, motility, metabolism, and gene regulation. Moreover, a fine balance within the MAPK signalling pathway is required as an important event in the maintenance of proper neural cell proliferation and differentiation during brain development. To assess the activation of MAPK (p-ERK1/2, p-p38 and p-JNK1/2) in NPCs induced by RV at various concentrations (1–50 μM) for 1 h, western blotting was performed. Data showed that RV (1, 10 and 20 μM) significantly increased the levels of p-ERK1/2 and p-p38 MAPK and p-CREB (cAMP response element-binding protein). These alterations were associated with an increase in anti apoptotic protein Bcl-2. While, higher dose of RV (50 μM) significantly decreased the levels of p-ERK1/2 and p-p38 MAPK and a parallel increase in the levels of activated caspase-3 was observed. In addition, no significant changes were observed in case of p-JNK MAPK in all exposure groups of RV except 50 μM (Fig. 8a,b). RV at 10 μM significantly increased the phosphorylation of TrkA receptor protein, with a parallel increase in expression of SIRT1. While, an increase in p75NTR was found to be associated with a higher dose of RV (50 μM) (Fig. 8c,d). The neuronal differentiation markers were also probed for their altered expression on exposure of RV for 1 h with a significant increase in levels of PSA-NCAM and synaptophysin. RV however could not significantly modulate the expression of NMDA-R2 at any concentration (Fig. 8e,f). Moreover, when NPCs were exposed to 10 μM concentration of RV for variable time periods (1–6 h), RV induced significant expression of p-ERK1/2 by 1 h and sustained up to 2 h and thereafter decreased gradually till 6 h. Expression of p-p38 was also increased within 1 h, and gradually decreased from 2 h to 6 h. RV exposure could not significantly modulate the expression of p JNK1/2 (Fig. 8g). RV also induced transient alterations in the levels of p-CREB and the downstream markers of neuronal differentiation viz., PSA-NCAM and Synaptophysin. RV increased the expression of p-CREB, PSA-NCAM and Synaptophysin by 1 h that sustained till 2 h, thereafter decreased gradually till 6 h (Fig. 8h).

### Activation of MAPK signalling seems essential for RV-induced proliferation of NPCs

**Figure 9:**
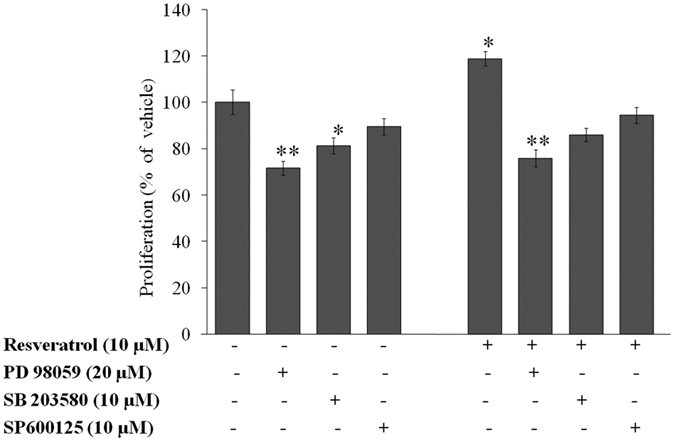
Resveratrol induced proliferation of NPCs via activation of ERK1/2 and p38 signalling molecules. Neural progenitor cells were treated with the indicated kinase inhibitors for 1 h with subsequent treatment with 10 μM Resveratrol for 24 h, and MTT assays were performed. Inhibitors used were: PD98059 (ERK inhibitor), SB203580 (p38 inhibitor), SP600125 (JNK inhibitor). Data represents mean ± SE of three independent experiments. *p < 0.05; **p < 0.01; ***p < 0.001 (Vehicle vs experimental group).

To examine whether activation of MAPK signalling is required for RV-induced proliferation in NPCs, cells were pre-exposed to specific inhibitors of MAPK such as PD98059 (ERK inhibitor), SB203580 (p38 inhibitor), SP600125 (JNK inhibitor) for 1 h and subsequent exposure to 10 μM RV for 24 h was given, followed by MTT assay. Results of MTT assay showed that pre-treatment with the inhibitors failed to induce proliferation of NPCs suggesting that these molecules are essential in RV-induced cell proliferation (Fig. 9). In addition, results also showed that in all these MAPK, ERK1/2 plays an important role to induce the proliferation of NPCs suggesting that this molecule was more essential in the action of RV-induced cell proliferation in NPCs. Moreover, these MAPK inhibitors significantly inhibited the proliferation of NPCs in the absence of RV suggesting that these molecules also play a significant role in the basal proliferation of NPCs.

### The proliferative action of RV is selective for neural progenitor cells

**Figure 10:**
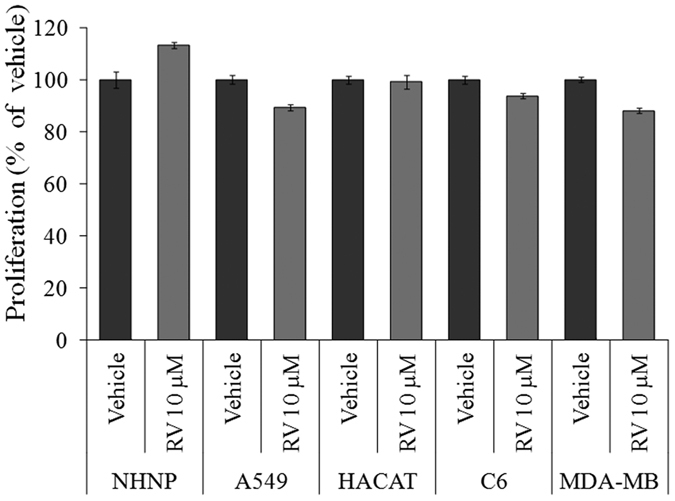
The proliferative action of Resveratrol is selective for neural progenitor cells. NHNP and the other cell lines (A549, human lung carcinoma; HaCaT, human keratinocytes cell line; C6, rat glioma cells; MDA-MB-231, human breast cell line;) were seeded into 96-well culture plates and cultured for 24 h. The cells were then exposed to 10 μM Resveratrol for 24 h, and cell proliferation was quantified using the MTT assay. Data represents mean ± SE of three independent experiments.

**Figure 11:**
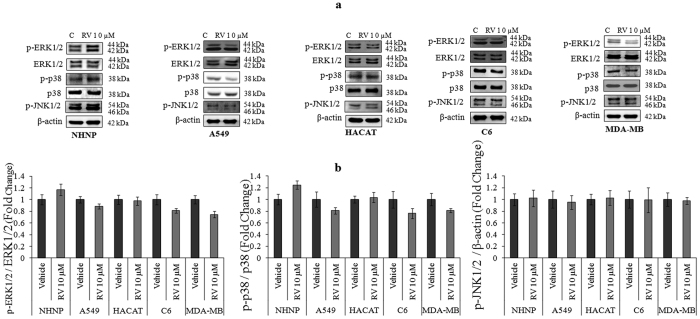
The proliferative action of Resveratrol is mediated through ERK1/2 and p38 signalling molecules activation. (**a**) Activation of the ERK1/2, p38 and JNK1/2 signalling molecules phosphorylation on exposure of 10 μM of Resveratrol for 1 h in NHNP and the other cell lines (A549, human lung carcinoma; HACAT, human keratinocytes cell line; C6, rat glioma cells; MDA-MB, human breast cell line;). (**b**) β-actin was used as an internal control to normalize the data. Quantification of proteins was done in Gel Documentation System (Alpha Innotech, USA) with the help of AlphaEaseTM FC Stand-Alone V.4.0 software and expressed in fold change.

**Figure 12:**
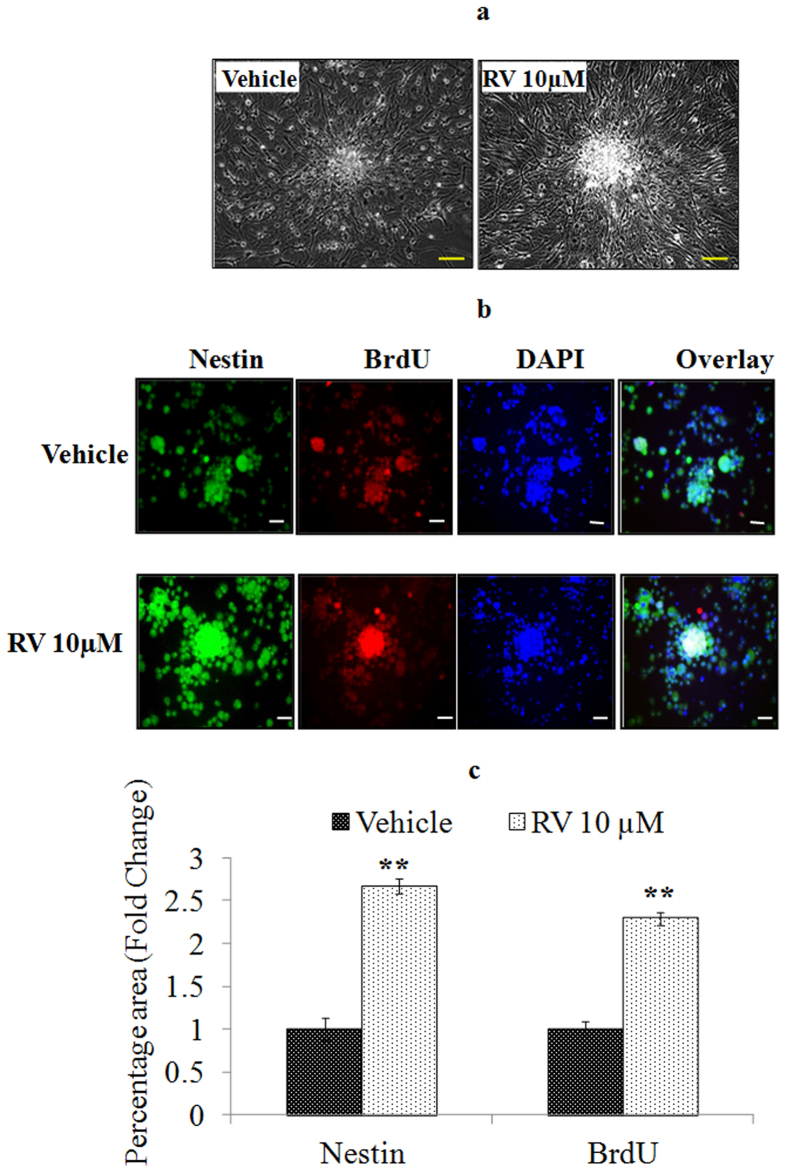
Resveratrol stimulates the proliferation of Normal human neural progenitor (NHNP) cells. (**a**) NHNPCs were cultured into 24-well culture plates and treated with Resveratrol (10 μM) for 48 h. Images were taken by Nikon DS-Ri1 (12.7 megapixel) camera at 20 × 10 magnification. (**b**) Representative photomicrographs showing Immunocytochemistry localization of neural progenitor cell marker viz. Nestin (green) and proliferative marker BrdU (red) in NPCs following the exposure of Resveratrol for 48 h. (**c**) Image quantification was done using ImageJ image analysis software and expressed in fold change *p < 0.05; **p < 0.01; ***p < 0.001.

To examine the effect of RV on the proliferation of several cells including human neural progenitor cells (hNPCs), normal human epithelial cells (HaCaT) and cancer cell lines including metastatic breast cancer cell line (MDA-MB-231), lung cancer cell line (A549) and rat glioma (C6) cells (1 × 10^4 ^cells/ml) were exposed to RV (10 μM) for 24 h. Results showed that RV (10 μM) only induced proliferation in hNPCs and its proliferative effect was not observed in other cells suggesting that proliferative action of RV is very selective for neural progenitor cells (Fig. 10). Further, western blotting was performed to investigate the activation of MAPK signalling in all the cell types exposed to RV (10 μM) for 1 h. Results showed that RV (10 μM) exposure activated MAPK signalling only in hNPCs and no activation was observed in any other cell type (Fig. 11a,b). The morphological and immunocytochemical results also confirmed the proliferative action of RV (10 μM) in hNPCs as observed by an increase in the size of the neurosphere and a significant upregulation of progenitor cell marker (Nestin) and proliferative marker (BrdU) on exposure for 48 h (Fig. 12). We also confirmed the involvement of ERK1/2, p38 and JNK1/2 MAPK molecules in RV-induced proliferation in hNPCs by using specific pharmacological inhibitors of these molecules (data not shown). These findings suggested that same pathways are involved in RV-induced proliferation in both hNPCs and rNPCs.

### RV induces neurogenesis in aged rats

**Figure 13:**
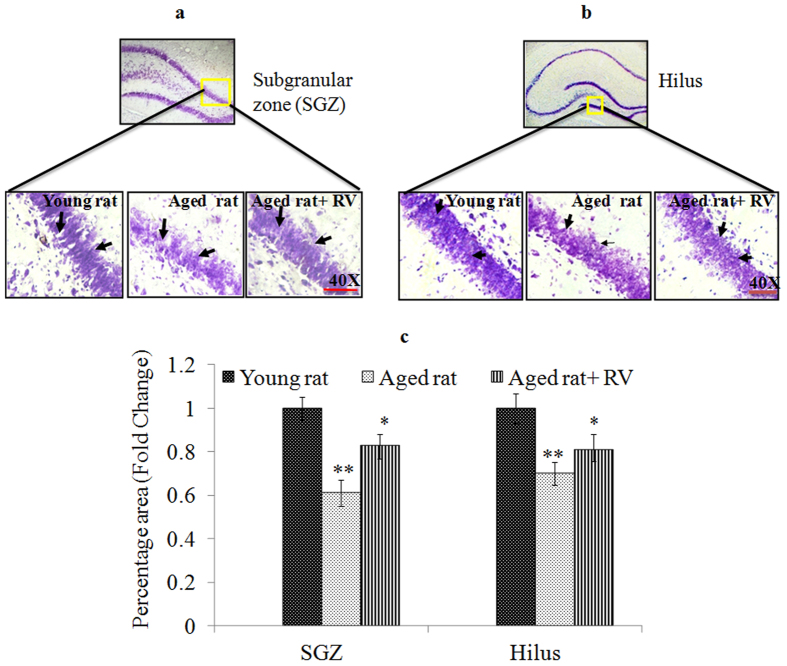
Resveratrol stimulates the proliferation in dentate gyrus area of hippocampus in aged rat. (**a**) Photomicrographs of SGZ region illustrating Nissl staining in young rat, aged rat and aged rat treated with Resveratrol (20 mg/kg, body weight, p.o.,) for 45 days. (**b**) Photomicrographs of hilus region illustrating Nissl staining in young rat, aged rat and aged rat treated with Resveratrol (20 mg/kg, body weight, p.o.,) for 45 days. (**c**) Data represents mean ± SE of three independent experiments. *p < 0.05; **p < 0.01; ***p < 0.001 and is expressed in fold change.

**Figure 14:**
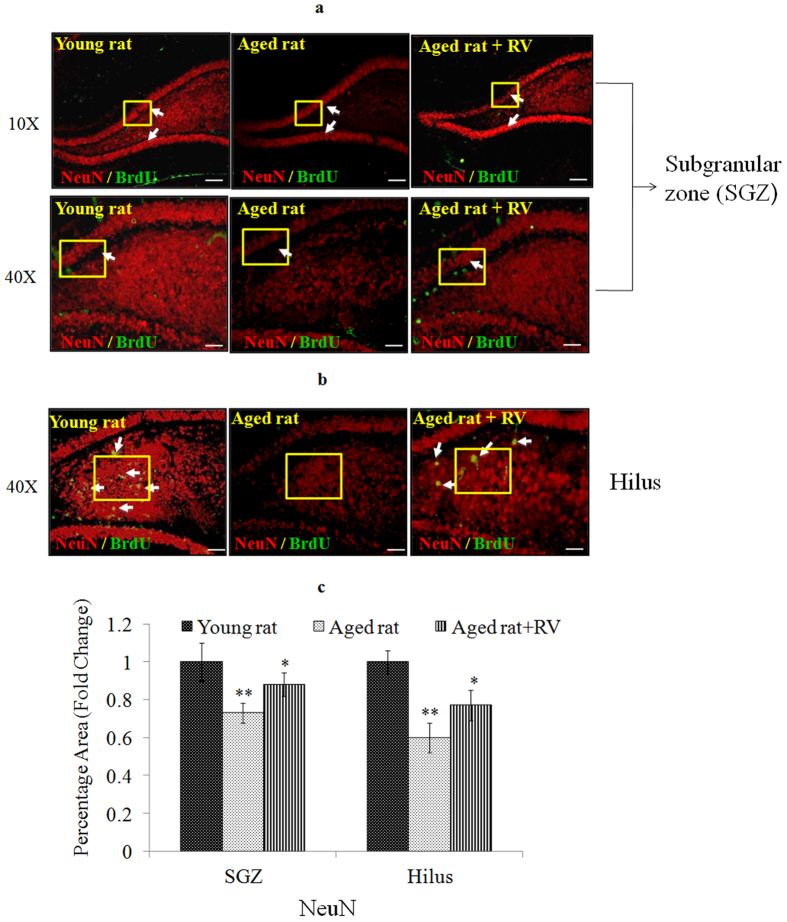
Resveratrol treatment increases the number of BrdU-positive cells in the Dendate gyrus area of hippocampus in aged rat. (**a**) Photomicrographs of SGZ region from young, aged and Resveratrol (20 mg/kg, body weight, p.o., for 45 days)-treated aged rats double labeled with fluorescent probes using antibodies against the mature neuron-specific protein (NeuN) and BrdU antibody. BrdU-positive cells (green, proliferation marker); NeuN-positive cells (red, mature neuron maker). **(b**) Photomicrographs of hilus region from young, aged and Resveratrol (20 mg/kg, body weight, p.o., for 45 days)-treated aged rats double labeled with fluorescent probes using antibodies against the mature neuron-specific protein (NeuN) and BrdU antibody. BrdU-positive cells (green, proliferation marker); NeuN-positive cells (red, mature neuron maker). (**c**) Data represents mean ± SE of three independent experiments. *p < 0.05; **p < 0.01; ***p < 0.001 and is expressed in fold change.

To determine whether RV induces neurogenesis in aged rats, Nissl staining was used in histological examination of hippocampal sections of RV (20 mg/kg body weight, p.o. for 45 days) treated aged rats. The data were compared to aged untreated control and young untreated control rats. Results of Nissl staining showed a significant decrease in the density of Nissl granules in the sub-granular zone (SGZ) of dentate gyrus area of hippocampus in aged rats (0.61 ± .04) as compared to young control rats. This decreased density of Nissl granules in the dentate gyrus area of hippocampus was found to be recovered significantly (0.83 ± .03) in rats treated with RV (20 mg/kg body weight, for 45 days) as compared to rats in aged control group (Fig. 13a,c). Based on this finding, we further confirmed the phenotypes of the newly generated cells in SGZ region of hippocampus region by NeuN/BrdU labelling. Results showed that more BrdU positive cells were observed in RV-treated aged rats and its number was higher when compared to aged control rats. These changes were also associated with a significant increase in the neurogenesis marker NeuN in aged rats treated with RV (0.88 ± .03) as opposed to rats in the aged group (0.73 ± .04) (Fig. 14a,c). These findings indicate that the treatment of RV promotes hippocampal neurogenesis in aged rats. Interestingly, apart from the known conventional zone of adult neurogenesis viz., sub-granular zone (SGZ) we also observed increased density of Nissl granules (control aged rats 0.7 ± 0.4 Vs aged rats treated with RV 0.81 ± 0.3) in areas overlapping the Hilus region and SGZ accompanied by an increase in BrdU positive cells and increased expression of NeuN (control aged rats 0.6 ± 0.4 Vs aged rats treated with RV 0.77 ± 0.3) as depicted in Figs 13b,c and 14b,c.

**Figure 15:**
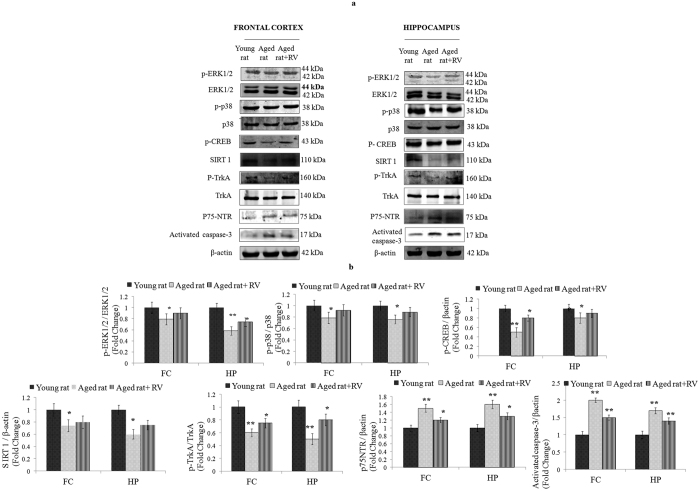
Resveratrol treatment induces the activation of ERK1/2 and p38 MAP kinases in fontal cortex and hippocampus regions of aged rats. (**a**) Protein expression profiling of p-TrkA, p-75NTR, activated caspase-3, MAPK, p-CREB and SIRT1 were studied in fontal cortex and hippocampus regions of young rats, aged rats and Resveratrol (20 mg/kg body weight, p.o., for 45 days) treated aged rats. (**b**) β-actin was used as internal control to normalize the data. Quantification of proteins was done in Gel Documentation System (Alpha Innotech, USA) with the help of AlphaEaseTM FC StandAlone V.4.0 software and expressed in fold change. *p < 0.05; **p < 0.01; ***p < 0.001.

TrkA signaling has been demonstrated to play a key role in the induction of proliferation and differentiation in NPCs during brain development^23^. We observed an increase in the phosphorylated levels of p-TrkA in aged rats administered with RV with a parallel decrease in p-75NTR, the latter activating death signalling pathways by inhibition of TrkA receptor^24^. These changes were associated with decreased levels of activated caspase-3 on administration of RV to aged rats. We also assessed the status of MAPK, p-CREB and anti-aging protein SIRT1 in fontal cortex and hippocampus brain regions of RV-treated aged rats and compared the data to aged control and young control rats. Results demonstrated significant inhibition of p-ERK1/2, p-p38, p-CREB and SIRT1 in the hippocampus of the aged rats. While no significant changes were observed in JNK MAPK in both brain region (data not shown). Although, the expression of p-ERK1/2, p-p38, p-CREB and SIRT1 was observed in both hippocampus and frontal cortex, the effect was more prominent in hippocampus region (Fig. 15a,b). Treatment of RV significantly increased the expression of p-ERK1/2, p-p38, p-CREB and SIRT1 in both hippocampus and frontal cortex regions in comparison to aged control rats.

## Discussion

One of the hallmarks of pharmacological science in medicine research is the development of safe drugs for the treatment of age related neurodegenerative disorders. Calorie restriction (CR) is well established to have a positive influence on neurogenesis, learning, and memory as well as cognition and mood^25^. As such, considerable research has been directed towards identifying substances that mimic the physiological effects of calorie restriction, and RV has emerged as a leading candidate in this realm. In the recent past, RV has also been the focus of numerous *in vitro* and *in vivo* studies investigating its wide biological attributes, which includes mainly anti-inflammatory, antioxidant activities, anti-atherogenic property, anti-platelet aggregation effect, oestrogen-like growth promoting effect, growth-inhibiting activity, immunomodulation and chemoprevention^26^,^27^,^28^,^29^. Moreover, it is hypothesized that the pharmacological properties of RV might have routed through some different mechanisms other than the conventional one^30^. In one of such studies, it has been observed that RV causes upregulation of ERK1/2 during retinoic acid induced differentiation in SHYSY5Y (human neuroblastoma cell line) cells^31^. Moreover, in other report, RV activated mitochondrial biogenesis and neurite out growth in Neuro2a cells via activation of AMP kinase pathway^32^. These studies have provided some initial clues regarding the neurogenesis potential of RV. However, the link of the activation of MAPK with neurogenesis are still missing and are to be explored because they are the key regulators for switching on/off the neural activity^33^,^34^. Here we extend this literature by successfully demonstrating that RV exhibits dose dependent discrepancies and at a lower concentration can have a positive impact on the proliferation, survival of NPCs and aged rat hippocampal neurogenesis implicating its potential as a therapeutic candidate for treatment of age related disorders.

The therapeutic mechanism of RV in the brain remains largely unclear. To gain insight into the biological effects of RV on neurogenesis, we examined its influence on NPCs. Our data reveal that RV enhances proliferation of embryonic NPCs and promotes neurogenesis in aged rat hippocampus. A concentration of 10 μM was found to be most effective in inducing proliferation of NPCs. However, concentration above 20 μM inhibited NPCs growth and was cytotoxic. Lower dose of RV significantly increased the size of neurospheres with a parallel increase in the expression of proliferation markers like Nestin and SOX2. MAPKs play a critical role in the regulation of cell growth and in the control of cellular responses to cytokines and stresses^21^,^22^,^35^. However, disturbances in MAPK cause tumorigenesis, neuron-inflammation and genetic and cellular alterations^36^. Infact mitogens like Epidermal Growth Factor (EGF) and basic Fibroblast growth factor (bFGF); required for sustained proliferation of NPCs; mediate their mitogenic effects via the MAPK cascade^37^,^38^. Reports demonstrate that the duration of signaling through MAPKs may hold the key to various outcomes of EGF and bFGF stimulation. Transient vs prolonged activation of the MAPK pathway has been closely associated, respectively, with a proliferation inducing vs a differentiation-promoting response to neurotrophin application^35^. Our data suggest that the stimulatory effect of low doses of RV on NPCs is mediated by transient activation of ERK and p38 MAPK via increased phosphorylation of TrKA receptor known to be involved in the induction of proliferation and differentiation in NPCs^23^. Pre-exposure to ERK and p38 inhibitors abrogated proliferation in NPCs demonstrating a requirement for these kinases in the biological effect of RV. Participation of these molecules was also confirmed at protein expression level via western blot analysis. These alterations were associated with increase in levels of p-CREB and SIRT1 mediating cell survival, plasticity^39^ and life span extension^40^ respectively. Resveratrol exposure at higher concentrations (50 μM) however inhibited the phosphorylation of TrkA and MAPK with a resultant downregulation in the expression of p-CREB and SIRT1. Simultaneously exposure to high concentration of RV also decreased the expression of the anti- apoptotic protein Bcl-2 with a parallel increase in the expression of death receptor p75 NTR and activated caspase-3 (hallmark of apoptosis). These alterations in TrKA/p75NTR signalling triggering the inhibitory cascades in response to higher concentration of RV are similar to our previous findings reporting molecular switching mechanism of TrKA/p75NTR in neuronal cells exposed to Monocrotophos, mediating death signalling pathways^41^. Interestingly, a transient increase in the levels of neuronal differentiation markers viz PSA-NCAM and synaptophysin was also observed as downstream targets of p-CREB on exposure of low dose of RV confirming its proliferation inducing Vs a transient differentiation-promoting effect on NPCs. These findings suggest the critical role of TrKA mediated MAPK signalling for inducing proliferative effect of RV on NPCs.

In the light of previous findings demonstrating chemopreventive role of RV in various cancer cell lines, we examined the modulating effect of low dose of RV (10 μM) on the growth of broad spectrum of cancer cell lines. Our results suggested that low dose of RV was not stimulatory for any of the cancer cell lines. Moreover, our studies have also suggested that the effect of RV on cell proliferation and MAPK signaling is selective for neural progenitor cells. This finding might be explained by the observation that RV is both a potent anti-cancer drug as well as a neurogenesis modulator.

Accumulating evidence suggests that neurogenesis is involved in many physiological and pathological conditions, such as learning and memory, mental disorders, and degenerative neurological diseases^42^. Adult hippocampal neurogenesis (AHN) is dynamically regulated by several factors, which are pathological conditions (e.g., stress, disease, injury), complex behaviours (e.g., learning), and environmental influences (i.e., exercise and diet), etc.^3^,^5^,^43^. However, there is a very close relationship between the onset of aging and the appearance of neurodegenerative disorders that can lead to irreversible injuries to the brain. Ageing is associated with a decreased AHN, and aged rodents display impaired learning and memory abilities^44^,^45^. Our histological studies demonstrate RV mediated recovery from cell loss in aged rats indicative of a protective effect of RV. RV administration to aged rats also significantly increased NeuN expression (selective neuronal marker) in the SGZ of hippocampus indicating increased hippocampal neurogenesis as compared to aged rats. Interestingly, quite contrary to the existing dogma of adult neurogenesis being restricted to certain discrete regions in the brain^46^, in our results, regions overlapping the SGZ and hilus were also observed to exhibit increased nissl staining density and higher NeuN expression on exposure of RV. This finding is of deep interest and whether or not adult neurogenesis could occur or be triggered in regions other than the known conventional ones could be an interesting subject for future investigations.

TrkA signaling has been demonstrated to play a key role in the induction of proliferation and differentiation in neural progenitor cells (NPCs) during brain development^23^. Infact the crosstalk between TrKA and p75^NTR^ ligand has a complex role in regulating neural survival and death that is dependent on its binding to ligands and co-receptors^24^. The p75^NTR^ exerted mechanisms of inhibition of TrkA have been suggested as one among the important factors accelerating neurodegenerative disorders^47^. The decrease in the TrkA expression and a parallel increase in the level of p75^NTR^ has also been reported during normal ageing as well as in Alzheimer disease^48^.

Our results demonstrate that RV administration to aged rats increased the phosphorylated levels of TrkA with a parallel decrease in the expression of p75NTR receptor as compared to aged rats. Previous studies have shown that ageing is marked by a progressive alteration in the MAPK signalling suggesting that the differential regulation of these pathways underlie a common age-associated mechanism^49^. Thus a fine balance between these signalling molecules is crucial for cell survival proliferation and differentiation. Classic signaling modules, such as the MAPK cascade have been identified as downstream cellular events induced by TrkA activation. Our results reveal that RV administration to aged rats significantly upregulated the phosphorylated levels of ERK, p-38 and CREB in both the hippocampus and frontal cortex regions. Such alterations were found to be associated with decreased levels of activated caspase-3 in aged rats administered with RV indicative of reduced cell death via apoptosis as compared to aged rats. The enhancement of MAPK signalling and hippocampal neurogenesis by RV documented in our studies is similar to the positive effect of exercise and environmental enrichment, suggesting the possibility that RV might also enhance hippocampal function^50^. RV also increased the expression of SIRT1 protein that has been strongly implicated in mediating cell survival, energy homeostasis and life extension in diverse species^40^,^51^ and has thus emerged as a therapeutic target for the treatment of age related degenerative diseases^52^. This effect of RV seems to mimic that of dietary calorie restriction, which is also associated with activation of SIRT1 proteins. Furthermore, it has been proposed that RV mimics numerous aspects of calorie restriction on energy metabolism and disease resistance in all eukaryotes tested to date and in most of them, the effect appears dependent on SIRT1^16^,^53^,^54^,^55^,^56^.

Recently a study carried out by Park *et al.*^56^ reported inhibitory effects of RV on NPCs and hippocampal neurogenesis. This study revealed that RV could not stimulate proliferation of NPCs and MAPK activation in NPCs. However, in this study maximum of the experiments were carried out with higher dose of RV (>50 μM). In addition, the study did not reveal the exact dose and duration of exposure of cells to RV for activation of MAPK. This study also demonstrated that treatment of RV to young mice (4-weeks old) reduced hippocampal neurogenesis and caused cognitive impairment, in association with activation of AMPK and inhibition of p CREB and BDNF in the hippocampus. The discrepancy occurred due to use of relatively young animals (only four weeks old) that have relatively high rates of neurogenesis and external supplementation may cause adverse effects. Thus, our present study and this previous study differ in several aspects including the doses of RV used, animal ages and study duration.

**Figure 16:**
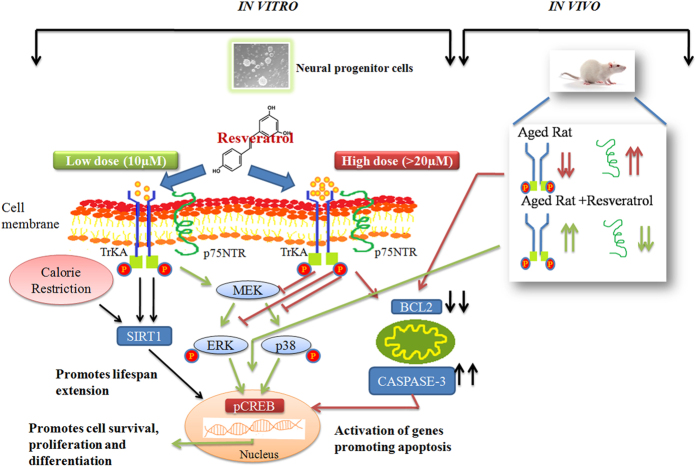
Resveratrol exerts dose specific biphasic responses. *In vitro*- At a low concentration (10 μM) Resveratrol activates the MAPK signalling pathway with a subsequent increase in the expression of p-CREB leading to proliferation, cell survival and differentiation. Resveratrol also independently activates SIRT1 proteins implicated in lifespan extension. However higher doses (>20 μM) inhibit the phosphorylation of the MAPK molecules with a parallel activation of activated caspase-3 inducing the apoptosis pathway. *In vivo*- The crosstalk between TrkA and p75^NTR^ ligand has a complex role in regulating neural survival and death. Classic signaling modules, such as the MAPK cascade have been identified as downstream cellular events induced by TrkA activation. In aged rats decreased phosphorylation of TrkA is observed with a parallel increase in the expression of the death receptor p75NTR leading to neuronal cell death. Administration of Resveratrol (20 mg/kg body weight) turns the molecular switch around with an increase in phosphorylated levels of TrkA with a subsequent increase in MAPK and p-CREB leading to cell survival and maintenance. This is an original figure made by one of the co-authors Ankita Pandey for publication with this manuscript only.

Our present study unequivocally shows that RV had a concentration-dependent stimulatory and inhibitory dual effect on the growth of NPCs (Fig. 16). Just like two sides of the same coin RV exhibits different cellular effects depending on the dose that is used. However, our present investigations strongly focuses on the stimulatory effects of lower concentration of RV i.e. 10 μM. In contrast to the above findings of Park *et al.* we further found an increase in the levels of p-CREB at lower dose of RV. Our data is again in sync with CR which also upregulates CREB^57^. This comparative analysis indicates that many effects induced by RV are dependent on dose and that opposite effects occur at low and high doses, being indicative of a hormetic dose response. In fact RV has shown to act in a hormetic-like biphasic dose/concentration response manner in numerous biological models^58^,^59^,^60^, its activity depending on many factors like the cell type, the organism being studied and the physiological and pathological state of the organism.

There is a flurry of research reports suggestive of RV having a large pharmacological window, however despite such progress, more research is needed to elucidate its mechanism of action. Also studies should focus on a wide range of concentrations using different cells in different experimental settings to understand its differential and contradictory cellular effects. Future studies of this striking form of adult plasticity will not only contribute to our understanding of the mechanisms and functional significance of neurogenesis in the adult mammalian brain but may also lead to novel strategies for cell-replacement therapy after injury and degenerative neurological diseases. It is fair to say that the literature on RV, in many cases, is contradictory and confusing. However, the body of evidence presented till date speaks volume for the potential of RV as a restorative therapy against age related disorders. In the meantime, we might all do well to follow the advice of Antonio Todde, once the world’s oldest man: “Just love your brother and drink a good glass of red wine every day”.

## Experimental Procedures

### Reagents and consumables

All the specified chemicals, reagents, diagnostic kits were purchased from Sigma Chemical Company Pvt. Ltd. St. Louis, MO, USA, unless otherwise stated. Culture medium, antibiotics, serum and growth factors were purchased from Gibco BRL, USA.

### Neural progenitor cells (NPCs) culture

NPCs were isolated from the embryonic day-12 (ED-12) rat foetuses. The tissue was dissected, washed with cold HBSS (Hanks Balanced Salt Solution), minced thoroughly and incubated in 0.1% trypsin for 30 min, then 10 min in DNase (40 μg/ml) at 37 °C. The tissue was then homogenized gently to obtain a single cell suspension. The cells were showing more than 95% viability as determined by trypan blue dye exclusion. The cells were plated at a density of 0.5 × 10^6^ viable cells/ml in 75 cm^2^ flasks in serum free neurobasal medium containing N-2 supplement (1%), B-27 supplement (2%), EGF (10 ng/ml), bFGF (10 ng/ml) and 1% antibiotic-antimycotic solution and allowed to grow as neurospheres at 37 °C in 5% CO_2_ and 95% air under high humid conditions. Medium was changed twice weekly, while fresh EGF and bFGF were added every day. Small proliferating neurospheres appeared after 1 week, which came to maturity by day 20. The neurosphere culture was passaged at an interval of 10–12 days by gently triturating the neurospheres and re-plating the single cell suspension of NPCs. The expanding NPCs were labelled with BrdU (5-Bromo-2′-deoxyuridine) (1.0 μM) for 24 h. To visualize the proliferating activity of NPCs and to identify the undifferentiated progenitor cells, neurospheres were co-immunostained with anti-nestin monoclonal antibody (mouse, 1:200; a neural stem and progenitor cell specific marker) and anti-BrdU antibody (rabbit, 1:500, proliferating cells marker).

### Other cells cultures

Primary human NPCs were obtained from Lonza Ltd, Basel, Switzerland (NHNP, PT-2599) and were cultured as per manufacturer’s instructions. Cancer cell lines including human lung carcinoma (A549) and human breast cell line (MDA-231) were grown in DMEM- F12 (Dulbecco’s Modified Eagle Medium-F12) and high glucose medium containing 10% fetal bovine serum, 0.2% sodium bicarbonate and antibiotic/antimycotic cocktail in a humidified atmosphere of 5% CO_2_, 95% air at 37 °C. HaCaT was also grown under identical conditions in RPMI-1640 supplemented with 10% fetal bovine serum, L-2 mM L-glutamine, while rat glioma cells (C6) were grown in above mentioned conditions in DMEM containing 10% fetal bovine serum, 5% horse serum, and 2 mM L-glutamine. For all studies, viability of the cells was measured by trypan blue dye exclusion, and batches having more than 95% cell viability were used in the study. Resveratrol (purity >99%) was dissolved in dimethyl sulfoxide (DMSO); an equivalent amount of DMSO was added to control cultures.

### MTT Assay

To examine the role of MAPKs in RV induced proliferation of NPCs, cells were pretreated with MAPKs inhibitors including PD98059 (ERK inhibitor, 20 μM), SB203580 (p38 inhibitor, 10 μM) and SP600125 (JNK inhibitor, 10 μM) for 1 h prior exposure to RV (10 μM) for 24 h. On completion of exposure time, the groups were processed for MTT (3-(4,5-dimethylthiazol-2-yl)-2,5-diphenyltetrazolium bromide) assay. In brief, cells (1 × 10^4^ cells/ml) were seeded in 96-well plates for 24 h under high humid environment in 5% CO_2_ -95% atmospheric air at 37 °C. The medium was aspirated and cells were exposed to variable concentrations of RV (1–100 μM) for 24–96 h. Tetrazolium bromide salt (10 μl/well; 5 mg/ml of stock in PBS) was added 4 h prior to the completion of respective incubation periods. Plates were incubated at 37 °C for 4 h, MTT solution was removed, and cells were lysed using a culture grade DMSO by pipetting up and down several times until the content was homogenized. After 10 min incubation, the color was read at 550 nm using multi-well microplate reader (Synergy HT, Bio-Tek, USA). The unexposed sets were also run simultaneously under identical conditions, which served as control.

### BrdU Immunocytochemistry

To demonstrate whether RV altered cell proliferation, the number of proliferative cells incorporated with BrdU (5-bromo-2′-deoxyuridine) was evaluated. Cells were labelled by adding BrdU to culture medium to a final concentration of 20 μM at 37 °C for 2 h, washed with BrdU-free culture medium, and exposed to either vehicle or RV. Twenty-four hours later, cells were fixed in PBS (pH 7.4) containing 4% paraformaldehyde, and washed with PBS. For immunostaining, fixed cells were post-fixed in 70% ethanol (in 50 mM glycine buffer, pH 2.0) at 20 °C for 20 min. DNA was denatured by sequentially exposing cells to heat (65 °C), acid (2 M HCl), and base (0.1 M borate buffer). Cells were then washed with PBS twice and incubated for 1 h in PBS containing 5% goat serum, 0.3% Triton X 100 and 2% BSA to block the nonspecific binding sites. Primary BrdU antibody (1:500) (Sigma) was then added, and plates were incubated overnight at 4 °C. Cells were then washed with PBS, incubated with secondary antibody, alexafluor^®^ 568 goat anti-mouse IgG (H+L) (1:400) (Life Technologies) and kept on a rocker shaker in dark for 2 h at room temperature. Cells were then washed with PBS three times for 5 min each and the cell nuclei was counterstained with 4′-6-diamidino-2-phenylindole (DAPI) followed by mounting with anti-fade (Invitrogen) cover slips. Thereafter, the cells were visualized under an upright fluorescence microscope (Nikon Eclipse 80i equipped with Nikon DS-Ri1 12.7-megapixel camera, Japan) using specific filters. For each marker, 20 randomly selected microscopic fields were captured and analyzed for fluorescence intensity with the help of ImageJ software analysis. The values are expressed in mean ± SE of percent area for fluorescence intensity covered.

### Immunocytochemical localization

Immunocytochemical localization was conducted following the protocol described^61^. After completion of the respective exposures, cells were fixed in 4% paraformaldehyde for 20 min. Cells were then washed with PBS twice and incubated for 1 h in PBS containing 0.02% Triton X 100 and 0.1% BSA to block the nonspecific binding sites. Cells were further washed with PBS and incubated overnight at 4 °C with primary antibodies against specific proteins, viz. Nestin (1:300) (Stem Cell Technologies) and Sox2 (1:400) (Cell signalling). Both the antibodies were diluted in PBS containing 0.02% Triton X 100 and 0.1% BSA. Following incubation with primary antibodies, cells were washed three times with PBS for 5 min each to remove the unbound antibodies. Then, Alexa Fluor 488 goat anti-mouse IgG (H+L) and Alexa Fluor 568 goat anti-rabbit IgG (H+L) secondary antibody were added to each well and kept on a rocker shaker in dark for 2 h at room temperature. Cells were then washed with PBS three times for 5 min each and the cell nuclei was counterstained with 4′-6-diamidino-2-phenylindole (DAPI) followed by mounting with anti-fade (Invitrogen) cover slips. Thereafter, the cells were visualized under an upright fluorescence microscope (Nikon Eclipse 80i equipped with Nikon DS-Ri1 12.7-megapixel camera, Japan) using specific filters. For each marker, 20 randomly selected microscopic fields were captured and analyzed for fluorescence intensity with the help of ImageJ software analysis. The values are expressed in mean ± SE of percent area for fluorescence intensity covered.

### Western Blotting

Western blot analysis was conducted following the protocol described earlier by us^61^. In brief, following respective exposures, cells were scraped, pelleted, and lysed using CelLytic M Cell Lysis Reagent (Sigma) in the presence of protein inhibitor cocktail (Sigma). After protein estimation by Bradford’s Reagent (Fermentas Inc., Glen Burnie, MD), equal amounts (40 μg/ well) of denatured proteins were loaded onto Tricine–SDS/ SDS-PAGE gel according to the protein molecular weight and blotted onto a polyvinylidene fluoride membrane (Millipore) by wet transfer method using transfer buffer (25 mM Tris [pH 8.3], 190 mM glycine, and 20% methanol) at 250 mA current for 2 h. Nonspecific binding was blocked with 2% BSA and 3% non fat dry milk powder in TBST (20 mM Tris-HCl [pH 7.4], 137 mM NaCl, and 0.1% Tween 20) for 2 h at 37 °C. After blocking, the membranes were incubated overnight at 4 °C with primary antibodies specific for p-ERK1/2, ERK1/2 p-p38, p38, p-JNK, p-CREB, SIRT1, TrkA, p-TrkA, P75NTR, PSA-NCAM, NMDA R2, Synaptophysin, Bcl-2, activated caspase-3 (1:1000, Chemicon) and β-actin (1:2000, Sigma) in blocking buffer (pH 7.5). The membrane was then incubated for 2 h at room temperature with secondary anti-primary immunoglobulin G (IgG)–conjugated horseradish peroxidase (Chemicon). The blots were developed using Super Signal West Femto Chemiluminescent Substrate (Thermo Fisher Scientific) and Bio-Rad Versa Doc Imaging System 4000 (Bio-Rad, Philadelphia, PA). The densitometry for protein-specific bands was conducted in Gel Documentation System (Alpha Innotech) with the help of Alpha Ease FC Stand Alone V.4.0 software.

### Animals and treatment schedule

The protocol for the study was approved by the Institutional Animal Ethics Committee of CSIR-Indian Institute of Toxicology Research (CSIR-IITR), Lucknow, India, and all experiments have been carried out in accordance with the guidelines laid down by the committee for the purpose of control and supervision of experiments on animals, Ministry of Environment and Forests (Government of India), New Delhi, India. Young (2 month) and aged (15 month) male rats of Wistar strain were obtained from the animal breeding colony of CSIR-IITR, Lucknow, India and housed in polypropylene cages under standard conditions with a 12 hours light / dark cycle (lights on at 8:00 AM) at temperature 25 ± 2 °C. The animals were fed pellet diet obtained commercially and water *ad libitum*. There were three treatment groups containing nine rats each, i.e. young vehicle control, aged vehicle control and aged treated with RV (20 mg/kg, body weight, p.o. suspended in 2% gum acacia) for 45 days. On completion of 24 h of last administration, a set of 3 rats from each treatment group was sacrificed by cervical dislocation and used for the western blotting studies. Brain was quickly removed and dissected into specific regions- frontal cortex and hippocampus following the procedure as described by Glowinski and Iversen^62^. Separate set of 3 rats was used to assess BrdU incorporation immunohistochemistry and Nissl staining following the procedure as described in detail. For that, rats were anaesthetised by injecting ketamine/xylaxine (37.5 mg/kg and 5 mg/kg body weight respectively) intraperitoneally and transcardially perfused with 0.9% NaCl solution followed by 4% paraformaldehyde (PFA) in 0.1 M phosphate-buffered saline (PBS). The brains were removed and immediately fixed in 10% PFA-PBS for 2 h, then immersed in 10%, 20% and 30% sucrose-PBS. Subsequently, serial coronal sections (20 μm in thickness) from each rat were cut by freezing microtome (Slee Mainz Co., Germany) and section were mounted on microscope slides and stored at −20 °C until use.

### Western Blotting

Translational expression changes in frontal cortex and hippocampus region of rat brain following the exposure of RV were assayed following the method described earlier by us^63^. The brain tissue was homogenized in RIPA buffer and further processed for western blot analysis. After protein estimation by Bradford’s Reagent (Fermentas Inc., Glen Burnie, MD), equal amounts (40 μg/well) of denatured proteins were loaded onto Tricine–SDS/SDS-PAGE gel and the rest of the procedure used was identical as described in the *in vitro* section. Expression levels SIRT1, p-ERK, ERK1/2 p-p38, p38 and p-JNK, p-TrkA, TrkA, p75NTR, activated caspase-3 (1:1000, Chemicon) and β-actin (1:2000, Sigma) were probed in these tissue samples.

### Histological studies

Nissl staining was used for histological examination of cell loss. Hippocampal sections from each treatment group were stained with cresyl violet (0.1%), dehydrated through graded series of alcohol and cover slipped with DPX mounting media^64^. The intensity of Nissl stained positive neurons in dentate gyrus area of hippocampal region was determined using a computerized image analysis system (Leica Qwin 500 image analysis software) as described by Shingo *et al.*^65^. The photomicrographs were processed on computerized software that enabled to assess the percent area of a selected field occupied by Nissl stained area.

### Immunohistochemistry (BrdU labelling)

To determine whether RV induces hippocampal neurogenesis in aged rats, BrdU immunohisto-chemistry was performed. Seven days before the end of the treatment period, BrdU (50 mg/kg body weight, i.p., 10 mg/ml freshly prepared in sterile 0.9% NaCl) was administered to all exposed groups once a day. For BrdU labelling, hippocampal sections of each group were post-fixed in 70% ethanol (in 50 mM glycine buffer, pH 2.0) at 20 °C for 20 min. DNA was denatured by sequentially exposing cells to heat (65 °C), acid (2 M HCl), and base (0.1 M borate buffer). Sections were then washed with PBS twice and incubated for 1 h in PBS containing 5% goat serum, 0.3% Triton X 100 and 2% BSA to block the non specific binding sites. Primary antibodies, anti-BrdU (1:500, Sigma) and anti-NeuN (1:200, Millipore) were then added, and sections were incubated overnight at 4 °C. Sections were then washed with PBS, incubated with secondary antibody, alexafluor 488 goat anti-mouse IgG (H+L) (1:400) and alexafluor 568 goat anti-mouse IgG (H+L) (1:400) (Life Technologies) and kept on a rocker shaker in dark for 2 h at room temperature. Slides were then washed with PBS three times for 5 min each and mounted with anti-fade (Invitrogen) cover slips. Thereafter, the cells were visualized under an upright fluorescence microscope (Nikon Eclipse 80i equipped with Nikon DS-Ri1 12.7-megapixel camera, Japan) using specific filters using ImageJ software analysis.

### Statistical analysis

Results are expressed as mean ± standard error of mean (SEM) for the values obtained from at least three independent experiments. Statistical analysis was performed using one-way analysis of variance (ANOVA) using Graph Pad Prism (Version 5.0) software. The values *p < 0.05 were considered as significant, **p < 0.01 more significant and ***p < 0.001 highly significant.

## Additional Information

**How to cite this article**: Kumar, V. *et al.* Differential responses of *Trans*-Resveratrol on proliferation of neural progenitor cells and aged rat hippocampal neurogenesis. *Sci. Rep.* **6**, 28142; doi: 10.1038/srep28142 (2016).
